# Opera trainees’ cognitive functioning is associated with physiological stress during performance

**DOI:** 10.1177/10298649231184817

**Published:** 2023-07-10

**Authors:** Negin Motamed Yeganeh, Taylor McKee, Janet F. Werker, Nancy Hermiston, Lara A. Boyd, Anja-Xiaoxing Cui

**Affiliations:** The University of British Columbia, Canada; The University of British Columbia, Canada; The University of British Columbia, Canada; The University of British Columbia, Canada; The University of British Columbia, Canada; University of Vienna, Austria

**Keywords:** music performance, opera performance, executive functions, physiological stress, heart rate variability

## Abstract

In an opera performance, singers must perform difficult musical repertoire at a high level while dealing with the stress of standing before a large audience. Previous literature suggests that individuals with better cognitive functions experience less stress. During a music performance such functions, especially attention, memory, and executive function, are in high demand, suggesting that cognitive functions may play a role in music performance. This study used physiological and cognitive measures to examine this phenomenon in opera performance. Cardiac activity data were collected from 24 opera trainees during a resting-state period before and during a real-life performance. Heart-rate variability (HRV) was used as an indicator of physiological stress, such that higher HRV indicates lower stress. Standardized neuropsychological tests were used to measure attention (IVA-2), memory (CVLT-3, WMS-IV), and executive function (Trail Making Test). Results showed cognitive function- and state-specific relationships between HRV and cognitive function: HRV during the resting state had a positive correlation with attention, while HRV during a performance had a positive correlation with executive function. These results suggest that greater cognitive function is related to lower stress during opera performance. The findings of this study provide initial evidence for a relationship between cognitive functions and music performance stress in opera trainees.

Opera is an intense art form, where many things happen at once. Opera trainees face many demands, from memorizing musical material, the texts of which are often in foreign languages, to drawing on their vocal technique when performing on stage. To meet these challenges, performers must exercise and engage attention, memory, and executive function (Lorusso & Porro, 2019). Visual and auditory attention are necessary for taking cues from the conductor and orchestra, and verbal and spatial memory are crucial for memorizing lyrics and stage direction. Executive functioning helps performers switch between tasks during a performance (Benz et al., 2016).

Research has shown links between cognitive functions and heart-rate variability (HRV), a metric derived from cardiac activity. In this work, higher HRV was associated with better cognitive functioning (Grol & De Raedt, 2020; Williams et al., 2019). HRV is a measure of the variation in the time interval between heartbeats (i.e., variability in the beat-to-beat interval). It is often used as an indicator of stress, such that lower HRV indicates higher stress (Castaldo et al., 2015; Pereira et al., 2017). As such, HRV can differentiate between musicians who are and who are not nervous before a performance (Harmat & Theorell, 2010), and between different phases of a music performance (Chanwimalueang et al., 2017; Williamon et al., 2013), including opera (Cui et al., 2022). Different HRV measures can be computed including time-based and frequency-based measures. Examples of time-based measures are the standard deviation of heart beat intervals (SDNN) and the root mean square of successive differences between normal heartbeats (RMSSD) (Kim et al., 2018; Singh et al., 2018).

Frequency domain analysis of HRV is used to measure the amount of relative or absolute signal energy detected in various frequency bands (Shaffer & Ginsberg, 2017). The high-frequency (HF) component is hypothesized to reflect the activity of the parasympathetic nervous system through the vagus nerve, while the low-frequency (LF) component reflects the activity of the sympathetic nervous system (Kim et al., 2018; Shaffer & Ginsberg, 2017).

Past research has demonstrated that higher HRV is associated with attention (Hansen et al., 2003; Siennicka et al., 2019; D. P. Williams et al., 2016), verbal (Gillie et al., 2014) and executive functions (Colzato et al., 2018; Frewen et al., 2013; Grässler et al., 2020; Hansen et al., 2003; Ottaviani et al., 2019; Thayer et al., 2009), but not visual memory (Forte et al., 2019; Shah et al., 2011). It is commonly concluded that individuals who experience less stress perform better on cognitive function tasks (Schaich et al., 2020; P. G. Williams et al., 2019).

A major issue in the field stems from the individual’s situation when HRV is being measured. Measurements taken during rest are viewed as characteristic of the individual and generalized across situations. On the contrary, measurements of HRV during a music performance illustrate changes in the individual’s state that can vary from moment to moment. As a result, there are two bodies of literature with different views on how cognitive functioning and stress are related. On one hand, results indicate that cognitive functioning worsens in stressful conditions (Frewen et al., 2013; Zeki Al Hazzouri et al., 2014) and, on the other hand, results indicate that people with better cognitive functioning experience less stress (Stawski et al., 2006).

In the present article, we report an investigation of the relationship between opera trainees’ cognitive functioning and HRV measured during both rest and live performance. We hypothesized that (a) concordant with previous research, trainees’ resting-state HRV is related to their cognitive functions, and (b) trainees with greater cognitive function at baseline experience less stress during a performance as indicated by higher HRV.

## Method

### Participants

Twenty-four opera trainees were recruited from three different opera workshops (6 males, 18 females, with a mean age of 23.41 years (*SD* = 4.03)). The Goldsmiths Musical Sophistication Index (Gold-MSI) self-report questionnaire (Müllensiefen et al., 2014) was used to further characterize the sample. Higher levels of musical sophistication would be generally characterized by a wider repertoire of musical behavior patterns and a higher frequency of behaviors demonstrating musical skills. Individuals with higher levels of musical sophistication should thus be able to respond to a greater range of musical situations when engaging with music. Values of the General Sophistication scale ranged from 90 to 123 (*M* = 105.00, *SD* = 7.91), one standard deviation higher than in the general public (Müllensiefen et al., 2014; see Table 2, p. 10, *M* = 81.58, *SD* = 20.62).

Participants were enrolled in several university opera programs. Three were on a program leading to a Diploma in Music Performance, 12 on a program leading to a Bachelor of Music, eight on a program leading to a Master of Music, and one was undertaking a PhD in music. At the time, they took part in the research they were preparing for performances of three operas (see below for details). The production of each opera was double cast. Opera trainees were not compensated for their performances. However, their performances were evaluated as part of their academic curriculum.

Seven participants were preparing for performances to be given in February 2021 of *Mansfield Park*, a two-act chamber opera composed by Jonathan Dove in 2011 with a libretto by Alasdair Middleton based on the novel by Jane Austen (1814). The plot and setting root the opera in the early 19th century in England and highlights the absurdity of human relations during this period. Sixteen participants were preparing for performances to be given in May 2021 and January/February 2022 of two different productions of *The Marriage of Figaro*, an *opera buffa* in four acts composed in 1786 by Wolfgang Amadeus Mozart with a libretto in Italian by Lorenzo Da Ponte. This is a story of love, revenge, and deception. One participant was preparing for a performance to be given in June 2022 of *Rusalka*, in three acts, by Antonín Dvořák with a libretto in Czech by Jaroslav Kvapil (1868–1950). The opera tells the story of a water nymph who falls in love with a mortal prince and exchanges her voice for legs so that she can be with him. Ethical approval for the study was sought and obtained from the University of British Columbia Behavioral Research Ethics Board (BREB) (certificate H19-03946).

### Data collection

#### HRV measurement

Participants were asked to arrive for data collection approximately 1 hour before the curtain was due to rise. Prior to data collection, participants were provided with written instructions and various instructional materials detailing how to properly fit and wear a Polar H10 sensor chest strap to measure HRV. The participants presumably placed the device on themselves according to the instructions provided, while a researcher was present to supervise the process. The Polar H10 chest strap uses an electrocardiogram (ECG) sensor to measure the electrical activity of the heart, which was used to calculate HRV. Data were collected twice during the study.

First, HRV was recorded before the start of the opera. Upon arrival at the performance center, participants were directed to proceed to the data collection room, which was a quiet environment. Participants were given a few minutes to settle in and place the recording devices on themselves, then asked to remain seated and still, breathing naturally, and avoiding physical activity before the resting-state data collection began. It should be acknowledged, however, that it was not feasible to control for any activity participants may have engaged in prior to the resting period. Although controlling for respiration is generally thought to be important (Laborde et al., 2017), there is evidence that respiration rate need not be controlled to obtain accurate HRV measurements (Denver et al., 2007). Second, HRV was recorded during the performance of the opera. The chest straps were paired with iPod touch devices, concealed in waist packs, and worn underneath costumes.

Participants were supervised by the researchers during the resting-state recording. The researchers time-stamped the beginning and end of the resting-state period, and the start and finish of the opera performance. Participants removed the chest straps when the cardiac activity data had been recorded in each condition and exported for analysis.

The HRV data were exported through the Elite HRV app, which uses a proprietary algorithm to process HRV data. This algorithm includes a moving average filter, detection of outliers, interpolation, detrending, and noise reduction techniques to remove artifacts from the time intervals between consecutive R-waves of the QRS wave complex given in milliseconds, termed R-R intervals. The QRS complex is a series of waveforms that represent the electrical activity of the ventricles of the heart during a heartbeat, typically consisting of a Q wave, an R wave, and an S wave. The R-R interval, also called the RR interval or the heart period, is the time interval between consecutive R-waves of the QRS complex and is commonly used in HRV analysis. By removing artifacts from the R-R intervals, the Elite HRV app ensures that the resulting HRV data are accurate and reliable (Elite HRV, 2020).

The root mean square of successive R-R intervals (RMSSD) is a commonly used marker of HRV modulated by the parasympathetic nervous system (Plews et al., 2013; Sherman et al., 2021). However, it is known to be influenced by external stimuli and the timing of measurement. Therefore, the standard deviation of R-R intervals (SDNN), which is not significantly influenced by breathing frequency, was selected as the primary indicator of HRV in the analysis. The code for calculating SDNN was modified from the open-source toolbox HRVTool (Vollmer, 2019). We used two metrics to represent HRV: *resting-state SDNN* and *SDNN opera*.

### Neuropsychological battery

Cognitive functioning was assessed using a battery of standardized neuropsychological assessments. All participants completed this battery during the 4-week period following the opera performance. Executive function was assessed using the Trail Making Task as implemented on a KINARM robot (Arbuthnott & Frank, 2000; Tombaugh, 2004). Different aspects of this task measure processing speed and task switching. As summary measure of executive function, we used the Trail B-Trail A score.

Attention was measured by the Integrated Visual and Auditory Continuous Performance Test (IVA-2; BrainTrain, 2020). This 13-minute test requires participant to click the mouse only when they hear or see the target (number 1) and not to click when they hear or see the nontarget item (number 2). As measures of attention, we used the summary scales Full Scale Attention, Sustained Attention, and Response Control.

Verbal memory was assessed using the California Verbal Learning Test (CVLT-3; Delis et al., 2017). CVLT-3 measures recall of two lists of words over a number of immediate and delayed memory trials. We used short-delay and long-delay free recall as measures of verbal memory. Immediate and delayed visuospatial memory were assessed using Designs I and Designs II from the Wechsler Memory Scale (WMS-IV; Wechsler, 2009).

Before we carried out the main analyses, we evaluated the potential influence of outliers on the outcomes. The Z-score method was employed to identify outliers, with participants whose Z-scores were greater than 3 in the neuropsychological assessment considered as outliers. Exploratory data analysis showed that the inclusion of outliers did not alter the results. We calculated correlation coefficients and compared the overall distribution of the data with and without outliers and found no statistically significant differences between them. Therefore, we included the outliers in our sample. Statistical analysis was conducted, both with and without these outliers, and the impact was analyzed through a comparison of the correlation coefficients and the overall distribution of the data.

## Results

This study was designed to investigate the relationship between HRV and cognitive function in a sample of 24 participants. It should be noted that a subset of participants was unable to complete the full cognitive battery, resulting in varying sample sizes for each relationship assessed and each relationship-specific analysis, with a potential impact on the representativeness of the sample population and the generalizability of our findings.

A Wilcoxon Signed-Rank test showed that SDNN was lower during the offstage resting period before the opera than on stage during the opera, *p* = .002 (resting-state SDNN: *M* = 64.51, *SD* = 22.55; SDNN opera: *M* = 92.65, *SD* = 38.22). There was no significant correlation between resting-state SDNN and SDNN opera, *p* > .05.

**Figure 1.**
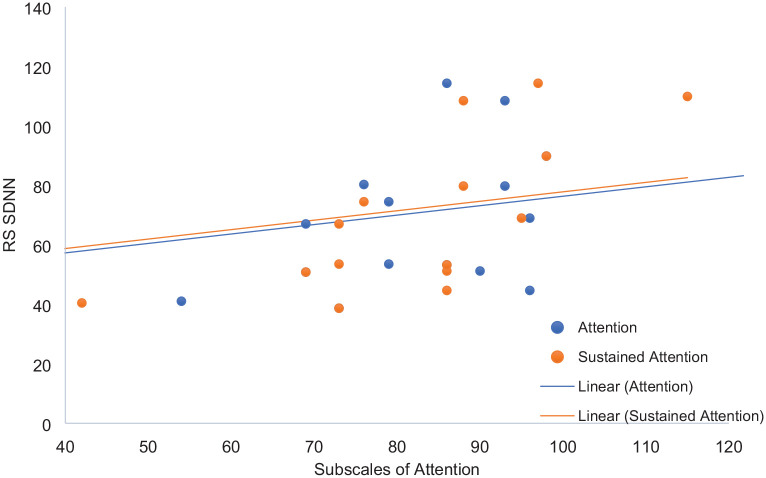
Overlay scatter diagram of resting-state SDNN and subscales of attention.

**Figure 2.**
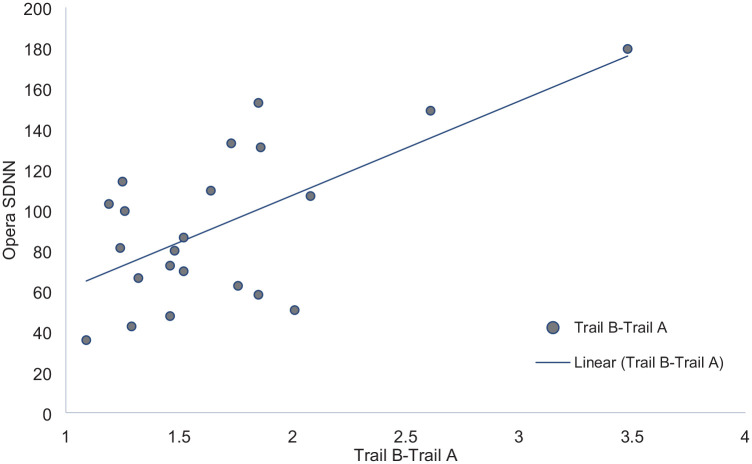
Overlay scatter diagram of Opera SDNN and executive function.

The association between the cognitive measures derived from the battery of standardized cognitive assessments and HRV was therefore explored using Spearman’s rank-order correlation. The analysis was performed separately for resting-state SDNN and SDNN opera. Resting-state SDNN correlated with two scales of attention: full scale, ρ_23_ = .51, *p* = .001 and sustained, ρ_23_ = .56, *p* = .005, see Figure 1. There were no significant correlations between resting-state SDNN and the measures of response control, verbal memory, and visual memory. SDNN opera correlated with measures of executive function: Trail total score, ρ_22_ = .55, *p* = .008, and Trail B-Trail A, ρ_22_ = .42, *p* = .049, see Figure 2. SDNN opera was correlated negatively with visual memory, Design I, ρ_22_ -.48, *p* = .021, Design II, ρ_22_ = -.46, *p* = .028. There were no significant correlations between SDNN opera, the measures of verbal memory, and attention. Neither resting-state SDNN nor SDNN opera correlated with general musical sophistication, *p*s > .05.

## Discussion

Concordant with our hypotheses, these results show that opera trainees’ resting-state HRV is related to aspects of cognitive function. Furthermore, trainees with higher cognitive functioning experience less stress as indexed by HRV measures during performance. Our results show that trainees’ resting-state HRV, recorded offstage before a performance, is related to their full-scale attention and sustained attention, and that greater executive function is related to a lesser experience of stress during a performance.

The findings of previous research (Appelgren et al., 2019; Grape et al., 2003) on the relationship between HRV and well-being during musical engagement emphasize the need to consider individual motivations and experiences. The relationship between cognitive function, performance anxiety, and HRV is complex and multifaceted, with the possibility of bidirectional relationships, such as the correlation between stage fright and anxiety during performance, and the impact of training investment on the relationship between cognitive function and HRV during musical performance. Appelgren et al.’s study found that intrinsic motivations have a greater impact on musical engagement, while Grape et al.’s study showed that the impact of singing on well-being differs between amateur and professional singers, with HRV increasing during singing in professionals but not in amateurs. These results highlight the complex nature of motivation and underscore the importance of further investigation into the bidirectional relationship between HRV and cognitive functioning.

The lack of connection between the literature on HRV’s relationship with cognitive functions and HRV as an indicator of musicians’ performance stress identified in the introduction is illustrated in our data. HRV measures obtained at rest and HRV measured during a task are not correlated with each other; furthermore, it appears that they relate to different measures of cognitive functioning. At the very least, our results indicate that resting-state HRV does not reflect HRV during a task.

A particularly important difference between resting-state HRV and HRV during performance relates to the potential influence of respiration. Singers must control their breathing during a performance; this in turn can influence HRV. This may account for the significantly higher SDNN observed during the opera but does not explain its positive association with executive function. It is also important to note the puzzling findings on visual memory. Although previous research has shown that HRV is related to verbal (Gillie et al., 2014) but not visual memory (Shah et al., 2011), we found no relation between HRV and verbal memory in our study, and a negative relationship between resting-state SDNN and visual memory.

The existing literature considering links between HRV and cognitive functioning is sparse. A recent review identified only 20 studies of HRV, each addressing particular aspects of cognitive functioning (Forte et al., 2019). Conflicting results may thus be due to different sample characteristics as well as procedural and analytical differences, for example differences in the types of HRV metrics used. We have previously pointed out that frequency-based measures may not be reliable in the context of music performance (Cui et al., 2022).

Future studies could include measures of anxiety at arrival to the event and stress prior to performance to obtain data on the level of perceived performance stress experienced by individuals. This would provide a clearer understanding of differences between performance stress and the experience of stress during a resting state period. Incorporating psychological assessments of anxiety in future studies would help to elucidate the differences between psychological and physiological stress.

### Limitations

This study is limited by the small size of the sample, and also because participants were trainees, which raises the question as to whether the findings can be generalized to professionals. The sample size also limits our ability to draw conclusions about associations between variables. Our results may not be applicable to other musicians because the demands on them vary with the conditions in which the performance takes place. Most of our participants were performing different roles in different operas, making it challenging for us to compare the measurements we took from them.

Although our pilot data (Cui et al., 2022) suggest that performers’ HRV while singing is not affected by the nature of the particular opera that is being performed, we plan to address this potential limitation in future studies by collecting data from a double cast in each opera, so that we can compare measurements taken from two performers playing the same role. Finally, while we know in great detail what participants did while they were onstage, we did not measure, and hence do not know, what they did while they were offstage. This should be controlled in future research.

## Conclusion

In conclusion, this study suggests a relationship between HRV and cognitive functioning in opera trainees. The specific nature of this relationship depends on the aspect of cognitive functioning measured and when HRV is assessed. Our results indicate that trainees who experience less stress during a resting period display greater attentional capacity than those who experience more stress. In addition, trainees with higher executive function show less physiological stress during an opera performance.

These findings provide the first evidence that cognitive functions may be related to performance stress in musicians. It is important to note that, while our study provides valuable insights into the relationship between HRV and cognitive functioning in opera trainees, the sample size is small, which limits the generalizability of our findings. However, our results could be used to inform future studies with larger sample sizes, helping to explore this relationship further, and its potential implications for musicians.
